# Impact of Oral Health on Quality of Life and Subjective Well-Being in Community-Dwelling Older Adults in Mexico

**DOI:** 10.1093/geroni/igaa057.731

**Published:** 2020-12-16

**Authors:** Irma Díaz, Neyda Ma Mendoza- Ruvalcaba, Elva Dolores Arias, Julio Diaz

**Affiliations:** 1 University of Guadalajara, Guadalajara, Mexico; 2 University of Guadalajara CUTONALA, Guadalajara, Jalisco, Mexico; 4 Hospital Civil Fray Antonio Alcalde, Guadalajara, Mexico

## Abstract

Objective: Associate the impact of oral health with quality of life and subjective well-being in the community-dwelling older adults in Mexico. Methods: Non-random sample; 326 subjects: age collected (60-69 / ≥ 70); gender (male / female); marital status (couple / no partner); schooling (0-6 years / ≥7); income for basic needs (yes / no); no depression (GDS-15), no cognitive impairment (MMSE) and comorbidity (no disease / ≥ 1 disease) to control biases. Oral conditions; Caries index (ICPOD) WHO criteria: Very low-Low; Moderate and High. Need for dental prostheses (WHO Manual): No prostheses needed (27-28 natural teeth or fixed / removable / total combination; Need prosthesis: 2-28 tooth without replacement. Xerostomia (Thomson Inventory); moderate to severe xerostomia > 17 points. Dependent variables: Quality of Life Related to Oral Health (GOHAI); 57-60 points: High perception. Subjective well-being: Moral Scale of the Geriatric Center of Philadelphia (PGCMS): Low score (0-11). Results: Age: 71.84 ± 7,278; female / male (70.9 / 29.1%). Controlling confounding factors, multiple logistic regression showed that the need for multi-unit or total prostheses; high CPOD index; severe xerostomia; and low perception of well-being subjective, were associated with low GOHAI scores: P = 0.000; P = 0.004; P = 0.003; P = 0.02 respectively. Subjective well-being only was associated with severe xerostomia and low CVRSO perception: P = 0.0 1; P = 0.02 respectively. Conclusion: Taking into account various confounding factors, the Quality of Life related to Oral Health was the most affected by the deterioration of oral health.

